# Pancreatic lymphangioma and concurrent intestinal lymphangiectasia in a dog

**DOI:** 10.1093/jvimsj/aalaf033

**Published:** 2026-02-11

**Authors:** Taisuke Ishikawa, Tomohide Kuramoto, Tatsuro Hifumi, Chika Kozuma, Machiko Kozuma, Keishi Hirano, Erisa Moriwaki, Shota Sakanoue, Noriaki Miyoshi, Naoki Miura, Osamu Yamato, Masashi Takahashi

**Affiliations:** Veterinary Teaching Hospital, Joint Faculty of Veterinary Medicine, Kagoshima University, 1-21-24, Korimoto, Kagoshima 890-0065, Japan; Veterinary Teaching Hospital, Joint Faculty of Veterinary Medicine, Kagoshima University, 1-21-24, Korimoto, Kagoshima 890-0065, Japan; Laboratory of Veterinary Histopathology, Joint Faculty of Veterinary Medicine, Kagoshima University, 1-21-24, Korimoto, Kagoshima 890-0065, Japan; M’s Animal Hospital, 15045-5, Nishinoomote, Kagoshima 891-3101, Japan; M’s Animal Hospital, 15045-5, Nishinoomote, Kagoshima 891-3101, Japan; Veterinary Teaching Hospital, Joint Faculty of Veterinary Medicine, Kagoshima University, 1-21-24, Korimoto, Kagoshima 890-0065, Japan; Veterinary Teaching Hospital, Joint Faculty of Veterinary Medicine, Kagoshima University, 1-21-24, Korimoto, Kagoshima 890-0065, Japan; Joint Faculty of Veterinary Medicine, Kagoshima University, 1-21-24, Korimoto, Kagoshima 890-0065, Japan; Laboratory of Veterinary Histopathology, Joint Faculty of Veterinary Medicine, Kagoshima University, 1-21-24, Korimoto, Kagoshima 890-0065, Japan; Veterinary Teaching Hospital, Joint Faculty of Veterinary Medicine, Kagoshima University, 1-21-24, Korimoto, Kagoshima 890-0065, Japan; Laboratory of Veterinary Clinical Pathology, Joint Faculty of Veterinary Medicine, Kagoshima University, 1-21-24, Korimoto, Kagoshima 890-0065, Japan; Veterinary Teaching Hospital, Joint Faculty of Veterinary Medicine, Kagoshima University, 1-21-24, Korimoto, Kagoshima 890-0065, Japan

**Keywords:** pancreas, endocrinology, lymphatic malformations

## Abstract

A 2-year-old Border Collie presented with watery diarrhea and weight loss. Laboratory testing disclosed hypoproteinemia and abdominal imaging identified striations suggestive of intestinal lymphangiectasia and a polycystic mass contiguous with the pancreas. Clinical signs were transiently ameliorated by using prednisolone at an anti-inflammatory dosage, but hypoproteinemia recurred accompanied by ascites within 1 month. Exploratory laparotomy identified pancreatic cysts and extensive inflammation of the duodenum and jejunum, and biopsy samples were taken for histopathology. The pancreatic cyst was lined by lymphatic vessels with no atypia and surrounding collagen fibers, highly suggestive of pancreatic lymphangioma. Histopathology of jejunal biopsy samples disclosed lipogranulomatous lymphangitis and lymphangiectasia. After the laparotomy, the dog was treated with prednisolone at a physiologic dosage and a low-fat diet, and remained asymptomatic for 1 year.

## Introduction

Lymphatic malformations, including lymphangiomas and other cystic variants, are well-characterized in human medicine, with established imaging features and histopathologic criteria.^1,2^ They typically are categorized as congenital anomalies of the lymphatic system and include both localized and systemic forms. In contrast, reports in veterinary medicine, particularly in dogs, remain sparse. Most cases in dogs involve cutaneous lymphangiomas,^1,3–11^ with only isolated accounts describing involvement of parenchymal organs such as the liver^12^ or spleen.^13^

Within the gastrointestinal tract, intestinal lymphangiectasia is a recognized manifestation of lymphatic dysfunction in dogs and is a major cause of protein-losing enteropathy (PLE). It is sometimes accompanied by lipogranulomatous lymphangitis, a poorly understood condition of suspected inflammatory or immune-mediated origin. Although these conditions are presumed to arise from developmental abnormalities of intestinal lymphatics, the broader context of systemic or congenital lymphatic malformations in dogs remains unclear.^14^^,^^15^ A single case of a generalized lymphatic anomaly in a dog has been documented in the literature.^16^

Here, we describe a dog with concurrent pancreatic lymphangioma, intestinal lymphangiectasia, and lipogranulomatous lymphangitis. This unique combination of findings suggests the possibility of a more widespread congenital lymphatic abnormality involving multiple organs. We also present the clinical course of the dog, including transient improvement with anti-inflammatory treatment and apparent resolution of clinical signs of PLE after partial resection of the pancreatic cyst.

## Case description

A 2-year-old neutered male Border Collie was referred to Kagoshima University Veterinary Teaching Hospital with a chief complaint of chronic diarrhea (grayish-white in color), progressive weight loss, and suspected PLE. The referring veterinarian had identified severe hypoglobulinemia (globulin, 18 g/L; reference interval [RI], 23-38 g/L) and hypoalbuminemia (albumin, 12 g/L; RI, 23-40 g/L; Table S1), along with abdominal transudative ascites (total protein concentration, 0.2 g/L). No proteinuria was observed on urinalysis. Serum trypsin-like immunoreactivity was 18.3 μg/L (RI, 5-35 μg/L). Serum cholesterol concentration was low, likely as a consequence of intestinal malabsorption and diarrhea (Table S1). Hyperechoic mucosal striations were observed on abdominal ultrasonography of the small intestine.

The dog was started on prednisolone (1.2 mg/kg PO q24h), along with berberine tannate (4.2 mg/kg PO q12h), clopidogrel (2.1 mg/kg PO q12h), and a commercially available low-fat diet (dry formulation of Gastro Intestinal Low Fat diet, Royal Canin Japon, Tokyo, Japan; estimated energy density, 3.5 kcal/g; estimated fat content, 1.6-2.8 g/100 kcal). Berberine tannate was used for its possible anti-inflammatory and antimicrobial properties. Diarrhea resolved the next day, and the dog was referred for further evaluation.

**Figure 1:**
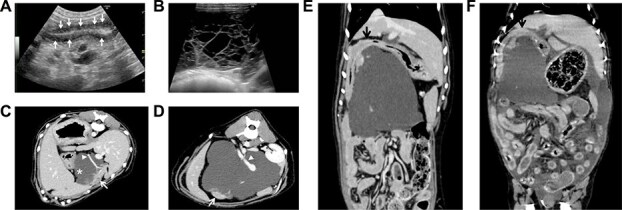
Imaging of jejunal and pancreatic abnormalities. (A) Abdominal ultrasonography of the jejunum showing striations, suggestive of intestinal lymphangiectasia. (B) Abdominal ultrasonography of the upper midline region showing a multicystic, fluid-filled lesion. (C) CT of the upper abdomen showing widely expanded pancreatic cysts (^*^) contiguous with the pancreatic parenchyma (arrow). Note the absence of visible septa within the cysts, which were observed on ultrasonography. The pancreaticoduodenal vein (arrowhead) is seen traversing the cysts. (D) Only a small remnant of the left pancreatic parenchyma (arrow), separate from the right pancreas (arrow shown in C), was observed. The portal vein (arrowhead) also was seen traversing the cysts. (E) A dorsal section on CT scan at the first admission identified a widely expanded cyst without compression of the duodenum (arrow). (F) A dorsal section at the re-admission showed that the cyst had moved to a more cranial position, compressing the duodenum (arrow) and impairing gastric emptying. Note the presence of ascites and fluid distension within the intestinal loops. Abbreviation: CT, computed tomography.

At referral, serum globulin and albumin concentrations had improved slightly (20 and 21 g/L, respectively), but ultrasonography still showed small intestinal striations (Figure 1A), ascites and multiple intra-abdominal cystic structures (Figure 1B). Fluid aspirated from one of the larger midline cysts had a markedly increased lipase concentration (>1000 U/L) while serum lipase concentration was 140 U/L, suggesting pancreatic origin. Computed tomography (CT) confirmed that this large cystic lesion was contiguous with the pancreatic parenchyma, and that the portal vein coursed between 2 distinct lobes, complicating complete surgical resection (Figure 1C-E). Contrast-enhanced CT imaging showed no clinically relevant enhancement within the cyst. Given the dog’s clinical improvement and decrease in ascites, endoscopic biopsy was not pursued, and the dog was discharged.

At follow-up 3 weeks later, the referring veterinarian reported further improvement in serum globulin (27 g/L) and albumin (27 g/L) concentrations with increases in appetite, activity, and weight. Two weeks later, despite continued prednisolone treatment, the dog experienced recurrence of hypoproteinemia, vomiting, and decreased appetite and activity. Upon readmission, serum globulin and albumin concentrations had decreased again (12 and 12 g/L, respectively) accompanied by marked ascites. Total serum lipase activity, measured using a catalytic method (Fuji Dri-Chem v-Lip-P Slide), was markedly increased (612 U/L; RI, 10-160 U/L), raising suspicion for acute pancreatitis. However, given the poor specificity of total lipase activity for diagnosing pancreatitis in dogs and the absence of canine-specific pancreatic lipase data, this finding was inconclusive.

Thoracic radiographs showed elevation of the diaphragm because of ascites, and contributing to tachypnea. Narrowing of the caudal vena cava and a small cardiac silhouette suggested decreased intravascular volume. Together with peritoneal effusion observed on ultrasonography, these findings indicated fluid shifts from the vascular space into the third space, likely caused by hypoalbuminemia-induced decrease in oncotic pressure. Non-contrast CT identified marked enlargement and cranial migration of the pancreatic cysts, which appeared to be compressing the duodenum and obstructing gastric outflow, leading to recurrent vomiting (Figure 1F).

**Figure 2:**
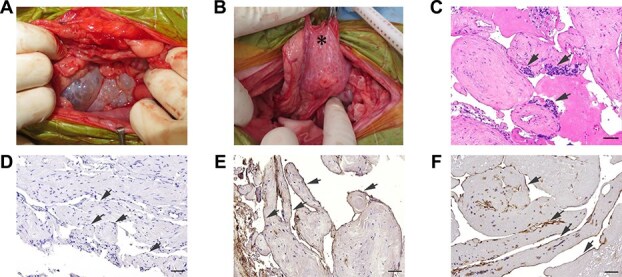
Macroscopic and histopathologic findings of pancreatic lymphatic abnormalities. (A, B) Macroscopic appearance of the pancreatic cysts. (A) Ventral pancreatic cysts showing relatively large, fluid-filled cysts. (B) Cysts contiguous with the pancreatic parenchyma exhibiting a smaller, spongiform appearance (^*^). (C) The cysts were composed of multiple irregularly dilated vessels lined with a single layer of endothelial cells having oval nuclei and spindle-shaped cytoplasm. These proliferative vessels showed mild atypia and no mitotic figures. Lymphocyte aggregation was observed in the interstitium (arrows). H&E stain. Bar = 50 μm. (D) Immunohistochemical labeling with pancytokeratin showed negative reactivity in the cystic wall (arrows). Bar = 50 μm. (E) Immunohistochemical labeling with von Willebrand factor showed diffuse, strong, cytoplasmic immunoreactivity in the endothelial cells lining the dilated vessels (arrows). Mayer’s hematoxylin counterstaining. Bar = 50 μm. (F) Immunohistochemical labeling with LYVE-1 showed diffuse, strong cytoplasmic immunoreactivity in the endothelial cells lining the dilated vessels (arrows). Mayer’s hematoxylin counterstaining. Bar = 50 μm. Abbreviations: H&E, hematoxylin and eosin; LYVE-1, lymphatic vessel endothelial hyaluronan receptor 1.

**Figure 3:**
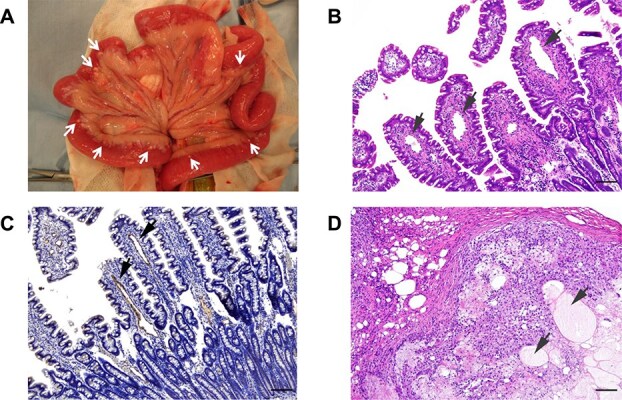
Macroscopic and histopathologic findings of the jejunum. (A) Intraoperative appearance of jejunum: white nodules were visible (arrows). (B) Multiple dilated lymphatic vessels in the lamina propria of the villi were observed (arrows). H&E stain. Bar = 100 μm. (C) Positive staining for LYVE-1 indicates multiple dilated lymphatic vessels. Bar = 100 μm. (D) Multiple disrupted lymphatic vessels filled with eosinophilic serous fluid in the muscularis layer were observed (arrows), and they were surrounded by lymphocytes, macrophages, epithelioid cells, multinucleated giant cells, and lipid vacuoles. H&E stain. Bar = 100 μm. Abbreviations: H&E, hematoxylin and eosin; LYVE-1, lymphatic vessel endothelial hyaluronan receptor 1.

Exploratory laparotomy was performed following a standard anesthetic protocol (atropine 0.02 mg/kg IV and propofol 4 mg/kg IV before intubation, followed by maintenance with sevoflurane inhalation anesthesia and a continuous rate infusion of fentanyl at 5 μg/kg/min). Macroscopic abnormalities were observed in both the pancreas and small intestine. The polycystic structures were predominantly relatively large sacs ventrally, but formed small spongiform cysts where they were directly adjacent to the pancreas (Figure 2A and B). The small intestine appeared edematous and thickened, with diffuse hyperemia and numerous small, white nodules primarily located at the junction of the bowel and mesentery (Figure 3A). Complete resection of the pancreatic cysts was not feasible. Therefore, full-thickness biopsy samples of the pancreatic cyst wall and the grossly abnormal jejunum containing a nodule were obtained for histopathological examination. During the procedure, the large pancreatic cyst was aspirated, so as to avoid damage to the portal vein traversing the cystic structures.

Histopathologic examination of the biopsied cystic wall identified multiple irregularly dilated and proliferative vascular channels lined by endothelial cells. These vessels were presumed to be lymphatic in origin, based on positive immunohistochemical staining for von Willebrand factor and lymphatic vessel endothelial hyaluronan receptor 1 (LYVE-1). The endothelial cells exhibited mild atypia without mitotic figures. Collagen fibers were observed within the septa of the lymphatic spaces, along with interstitial lymphocyte aggregation. Only minimal inflammatory cell infiltration was present in the cystic wall (Figure 2C-F). These findings were consistent with a diagnosis of lymphangioma.

Histopathology of the jejunum biopsy identified multiple dilated lymphatic vessels in the lamina propria of the villi. In the muscularis layer, disrupted lymphatic vessels filled with eosinophilic serous fluid were surrounded by lymphocytes, macrophages, epithelioid cells, multinucleated giant cells, and lipid vacuoles. These findings were consistent with lymphangiectasia and lipogranulomatous lymphangitis (Figure 3B-D).

**Figure 4:**
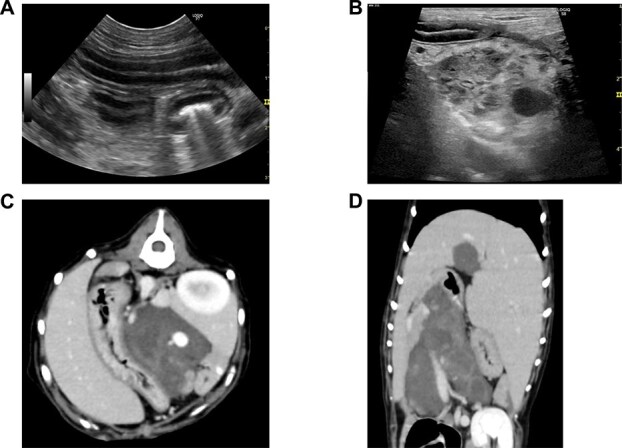
Postoperative imaging of the pancreatic cysts. (A) Abdominal ultrasonography of the jejunum showing resolution of the previously observed striations. (B) Abdominal ultrasonography showing marked decrease in size of the larger cysts, with the remaining smaller cysts exhibiting a spongiform appearance. (C, D) Follow-up CT scans showing a decrease in size of the pancreatic cysts. The transverse scan (C) 7 days postoperatively shows a substantial decrease in cyst size, with a small hyperdense component remaining, suggestive of scarring. The dorsal scan (D) shows further decrease in cyst size and absence of duodenal compression.

After laparotomy and partial cyst resection, the dog’s appetite improved, and both hypoglobulinemia and hypoalbuminemia resolved (Table S1), despite withholding prednisolone for 7 days. At 7 days postoperatively, imaging indicated a decrease in cyst size and resolution of intestinal striations on abdominal ultrasonography (Figure 4A and B). Computed tomography indicated relief of the duodenal compression and the presence of a small, hyperdense intracystic structure consistent with scarring at the biopsy site (Figure 4C and D). The dog was again started on a regimen of prednisolone (1.0 mg/kg/day) and a homemade ultra-low-fat diet (chicken filet and potato with an estimated fat content of 0.5 g/100 kcal). Over the first postoperative month, serum globulin and albumin concentrations remained within the normal range (24 and 40 g/L, respectively) and fecal consistency gradually improved from diarrhea to soft, near-normal feces.

During the year after surgery, the dog was changed onto a commercially available dry formulation of Gastro Intestinal Low Fat with fat content of 1.6-2.8 g/100 kcal, and the prednisolone was decreased to a maintenance dosage of 0.1 mg/kg/day. Abdominal ultrasonography confirmed the presence of small polycystic structures, which were less extensive than observed before the laparotomy. This decrease in cyst size likely was a result of cyst wall resection and possible inflammation induced by fluid aspiration.

## Discussion

The dog described in our report showed gastrointestinal signs and PLE, and was diagnosed with both pancreatic lymphangioma and intestinal lymphangiectasia. Lymphangiomas are rare, benign lymphatic malformations typically reported in the skin of dogs and in cervical or axillary tissues in humans.^1–11^ Although extremely rare, pancreatic lymphangioma may be included as a differential diagnosis for pancreatic cystic lesions, particularly in cases lacking evidence of pancreatitis or malignancy, as demonstrated in our case.

Clinically, the dog exhibited no vomiting at the time of referral, and serum lipase concentrations were not increased—findings inconsistent with pancreatitis. The pancreatic cystic lesion was clearly visualized on CT, which identified no signs of neovascularization or malignancy, and no evidence of systemic bone lesions, thereby excluding Gorham’s disease. Histopathologic evaluation of the cyst wall identified no inflammatory cell infiltration, further supporting the absence of pancreatitis. Immunohistochemically, the lesion was negative for pancytokeratin and positive for LYVE-1, suggesting lymphatic origin. However, because LYVE-1 also can be expressed in certain nonlymphatic cells, such as macrophages or vascular endothelial cells, these findings should be interpreted with caution.

Whether the pancreatic lymphangioma contributed to the development of intestinal lymphangiectasia remains uncertain. The resolution of hypoalbuminemia and clinical improvement after partial resection suggest a potential link, but co-occurrence could also be coincidental. Subtle lymphatic abnormalities in the mesentery or intestine may have been present but undetectable on imaging. Thus, pancreatic lymphangioma cannot be directly linked to PLE in this case, but the presence of extraintestinal lymphatic lesions should be considered in dogs presenting with PLE.

Our case, along with a previously reported Pomeranian dog with congenital lymphangiomas in multiple organs,^16^ emphasizes the potential for systemic or multifocal lymphatic malformations in veterinary patients. Our case also bears similarity to a report in which thoracic duct obstruction caused secondary lymphangiectasia in the mesentery and liver of a human patient.^17^ These findings raise the possibility of an underlying generalized lymphatic abnormality rather than isolated lesions. Continued monitoring of such patients may identify broader involvement.

One hypothesis is that the large pancreatic cyst mechanically impaired lymphatic flow, for example, by compressing the cisterna chyli, thereby increasing intralymphatic pressure.^18^ Alternatively, the cyst may have represented a blind-ending lymphatic structure, disrupting drainage. Both mechanisms may have contributed. Lack of inflammatory signs and localization of high lipase activity to the pancreas and cyst argue against enzyme leakage as a cause.

The combination of dietary fat restriction and immunosuppressive treatment initially improved clinical signs. However, recurrence of lymphangiectasia suggests that persistent cystic stasis may have perpetuated the condition. Partial cyst resection and drainage may have disrupted local stasis or induced remodeling through inflammation, similar to alcohol injection treatment used for pancreatic cysts and liver abscesses.^18,19^ Although symptomatic improvement was achieved, the risk of recurrence remains if systemic lymphatic abnormalities persist.

In conclusion, our report of concurrent pancreatic lymphangioma and intestinal lymphangiectasia with lipogranulomatous lymphangitis in a dog provides information for clinicians evaluating similar cases. Despite its rarity, pancreatic lymphangioma may be considered among the differential diagnoses for pancreatic cystic lesions in young dogs lacking signs of pancreatitis or malignancy. The coexistence of multiple lymphatic abnormalities raises the possibility of an underlying congenital lymphatic disorder not readily detectable on imaging. Additional studies are needed to clarify the relationship between localized and systemic lymphatic malformations in veterinary patients.

## Supplementary Material

aalaf033_supp_tab_1

## Off-label antimicrobial declaration

The authors declare no off-label use of antimicrobials.

## Institutional animal care and use committee or other approval declaration

The authors declare no institutional animal care and use committee or other approval was needed.

## Abbreviations

PLE: protein-losing enteropathy
CT: computed tomography
RI: reference interval
